# Evaluation of a new droplet digital PCR for diagnosis of pulmonary tuberculosis and tuberculous pleurisy

**DOI:** 10.3389/fmed.2026.1819767

**Published:** 2026-04-15

**Authors:** Xichao Ou, Huiwen Zheng, Xundi Bao, Peilei Hu, Zhou Liu, Jingwei Guo, Dongfang Xu, Yue Li, Jingjing Li, Bing Zhao, Jie Kang, Qi Ma, Hui Xia, Yunhong Tan, Yanlin Zhao

**Affiliations:** 1 National Key Laboratory of Intelligent Tracking and Forecasting for Infectious Diseases, National Center for Tuberculosis Control and Prevention, Chinese Centre for Disease Control and Prevention, Beijing, China; 2 Beijing Key Laboratory of Pediatric Respiratory Infection Diseases, Key Laboratory of Major Diseases in Children, Ministry of Education, National Clinical Research Center for Respiratory Diseases, Laboratory of Respiratory Diseases, Beijing Pediatric Research Institute, Beijing Children’s Hospital, Capital Medical University, National Center for Children’s Health, Beijing, China; 3 Department of Clinical Laboratory, Anhui Chest Hospital, Hefei, China; 4 Anhui Provincial Center for Disease Control and Prevention, Hefei, China; 5 Department of Clinical Laboratory, Hunan Chest Hospital, Changsha, China

**Keywords:** diagnosis, digital droplet PCR, pulmonary tuberculosis, tuberculous pleurisy, Xpert MTB/RIF

## Abstract

**Introduction:**

Digital droplet PCR (ddPCR) offers high sensitivity and absolute quantification without standard curves, making it promising for tuberculosis (TB) detection in paucibacillary samples. This study evaluated the diagnostic performance of a ddPCR assay targeting IS6110 and 23S rRNA for pulmonary tuberculosis (PTB) and tuberculous pleurisy (TP).

**Methods:**

A total of 436 patients (245 suspected PTB, 191 suspected TP) were prospectively enrolled. ddPCR was performed on sputum, bronchoalveolar lavage fluid, and pleural effusion samples. Diagnostic performance was compared with Xpert MTB/RIF and culture.

**Results:**

In suspected PTB, ddPCR showed significantly higher sensitivity than Xpert (49.70% vs. 38.10%, *p* < 0.001), while Xpert showed higher specificity (98.65% vs. 87.70%). In suspected TP, ddPCR also demonstrated superior sensitivity (17.54% vs. 10.40%, *p* < 0.001). For NTM detection, ddPCR achieved 60% sensitivity and 99.58% specificity.

**Discussion:**

ddPCR offers improved sensitivity for PTB and TP diagnosis, particularly in paucibacillary cases, though its lower specificity necessitates confirmatory testing. The dual-target design enables simultaneous MTB/NTM differentiation, providing clinical utility for guiding appropriate therapy.

## Introduction

Tuberculosis (TB) remains a leading cause of death from an infectious disease, with approximately 10.6 million cases reported worldwide in 2022, posing a serious threat to the global public health (1). Though great efforts have been made, China continues to rank third in a variety of TB burden indices globally (1). In addition to pulmonary tuberculosis (PTB), *Mycobacterium tuberculosis* (MTB) can also lead to extrapulmonary tuberculosis (EPTB). As the most common form of EPTB, tuberculous pleurisy (TP) accounts for approximately 50% of all EPTB patients in China (2, 3). If not diagnosed and treated promptly, TP can lead to serious complications, severely affecting patients’ health and quality of life (4, 5).

Conventional mycobacterial culture, the gold standard for TB diagnosis, is time-consuming, while acid-fast bacilli (AFB) smear has from low sensitivity (6). Molecular techniques have overcome some of these limitations and are now widely used for rapid diagnosis of MTB. The Xpert MTB/RIF assay (Xpert), in particular, has been recommended by the World Health Organization (WHO) for confirming of PTB and EPTB (7–9). However, due to the paucibacillary nature of TP, current microbiological and molecular tests show poor sensitivities for definite diagnosis, and precise quantification remains challenging (5, 10, 11).

Digital droplet PCR (ddPCR), a third-generation PCR-based technology, involves distributing nucleic acids from a sample to tens of thousands of separate reaction units, amplifying the nucleic acid molecules independently. By collecting and measuring the fluorescence signal from each reaction unit, the absolute copy number of the target nucleic acid can be accurately determined without the need for standard curves (12). This technique offers high tolerance to inhibitors, great precision and accuracy, making it particularly suitable for detecting low-abundance MTB infection (13, 14). In this study, we used two MTB specific targets, IS*6110* and *23S rRNA*, to evaluate the clinical diagnostic ability of ddPCR in PTB and TP, with the aim of improving diagnostic accuracy for PTB and TP.

## Methods

### Study participants

Patients with suspected PTB or TP were prospectively enrolled at Anhui Chest Hospital and Hunan Chest Hospital between May 2022 and May 2023. Inclusion criteria for suspected PTB were defined as the presence of one or more of the following clinical symptoms: persistent cough for ≥2 weeks (with or without sputum production), hemoptysis, chest pain, unexplained fever, night sweats, or weight loss, accompanied by chest radiographic abnormalities suggestive of tuberculosis. Additionally, an adequate sputum specimen of sufficient volume and quality must be available for testing. Inclusion criteria for suspected TP included clinical symptoms consistent with pleurisy (e.g., fever, cough, chest pain, dyspnea) and chest imaging findings suggestive of pleural effusion, along with the availability of an adequate pleural effusion specimen of sufficient volume and quality. All participants provided written informed consent after receiving a thorough explanation of the study’s purpose and significance. Exclusion criteria were as follows: (1) receipt of anti-tuberculosis treatment for more than 2 weeks prior to enrollment; (2) a previous history of TB diagnosis or treatment; or (3) inadequate specimen quality, including samples with insufficient volume or significant contamination.

### Clinical categories of participants

A clinically diagnosed case of PTB or TP was defined as a patient who did not meet the criteria for bacteriological confirmation, whose biological specimen is negative by smear microscopy, culture or WHO-approved rapid diagnostics (WRD), but was diagnosed with active disease by a clinician and subsequently initiated on a full course of anti-tuberculosis treatment. This definition includes cases diagnosed on the basis of X-ray abnormalities or suggestive histology without laboratory confirmation (15). In cases where clinical, radiological, and microbiological findings were discordant, the final diagnosis was determined by consensus among three senior clinicians based on a comprehensive review of all available data, including treatment response and follow-up outcomes. The non-TB was defined as cases with other diagnosis. The diagnostic pathway and classification criteria are summarized in Supplementary Table S1. The clinical information, including gender, age, clinical diagnosis, and examination results, were collected retrospectively.

### Laboratory study

Sputum or bronchoalveolar lavage fluid (BALF) was collected from suspected TB patients, and pleural fluid (PF) was collected from suspected TB pleurisy patients. All samples were promptly sent to the laboratory for the above routine clinical tests. For mycobacterial culture, specimens digested in L-cysteine NaOH-Na citrate (1.5% final concentration) and neutralized with phosphate buffer (PBS, 0.067 mol/L, pH = 7.4), were inoculated in Bactec MGIT 960 system for 6 weeks for mycobacterial culture. Positive cultures were subjected to Ziehl-Neelsen staining, and para-nitrobenzoic acid/thiophene-2-carboxylic acid hydrazide (PNB/TCH) used to distinguish MTB from non-tuberculous mycobacteria (NTM). The final reference standard for NTM diagnosis was determined by culture and Meltpro assay. For the Xpert MTB/RIF assay, 1 mL of sputum specimen was mixed with 2 mL of sample reagent, incubated at room temperature for 10 min, then transferred into a cartridge and loaded into the GeneXpert instrument. For bronchoalveolar lavage fluid and pleural effusion specimens, an initial centrifugation step was performed at 3000 × g for 15 min, after which the supernatant was discarded and the remaining sediment was mixed with 2 mL of sample reagent for subsequent processing as described above. The remaining specimens were used to extract DNA in batches using DNeasy Blood and Tissue Kits (Qiagen, Hilden, Germany) following the manufacturer’s instructions. The DNA samples were stored at −80 °C for ddPCR assay.

### ddPCR testing

ddPCR was performed using the Droplet Digital PCR System (Pilot Gene Technologies, Hangzhou, China), which comprises a DG32 droplet generator, a TC2 thermal cycler for droplet amplification, and a CS5 droplet reader. Three sets of primers and probes were designed for the detection of MTB and NTM. For MTB detection, primers targeting the *IS6110* element: (forward: 5′-GCTCAACGCCAGAGACCAG-3′; reverse: 5′-CCGGTGAGTCCGGAGACTC-3′) and the *23S rRNA* gene (forward: 5′-GTTGTAAGTTTTCGGCCGGTT-3′; reverse: 5′-TAGGTCAGACCTGGAAGCTCAGT-3′) were used, with corresponding MGB probes labeled with FAM (*IS6110* probe: 5′-CTGAGGTCTCAGATCAG-3′; *23S rRNA* probe: 5′-CACCAGTTCCCTACACC-3′). For NTM detection, a primer pair targeting a conserved region (forward: 5′-TGGGTCTAATACCGGATAGGACC-3′; reverse: 5′-AAGCTGATAGGCCGCGG-3′) was used, along with an MGB probe labeled with ROX (5′-CCGCAAAAGCTTT-3′). The total volume of the ddPCR reaction was 15 μL, consisting of 10 μL ddPCR reaction solution and 5 μL of extracted nucleic acid sample. Then, the 14 μL mixture was placed in a microfluidic chip and loaded into the DG32 droplet generator for droplet generation.

The amplification protocol consisted of an initial incubation at 50 °C for 10 min, denaturation at 95 °C for 2 min, followed by 45 cycles of 95 °C for 10 s and 60 °C for 20 s. After thermal cycling, the chip was transferred to the CS5 droplet reader for automated fluorescence signal measurement. The concentrations of the DNA were calculated using GenePMS Version 1.0.1 (Pilot Gene Technologies). The FAM channel was used to detect *Mycobacterium tuberculosis* complex, including *M. tuberculosis*, *M. bovis*, *M. microti*, and *M. africanum*. A sample was considered positive for MTB when the FAM channel detection value is greater than 0.46 copies/μL. The ROX channel was used to detect 16 common NTM subtypes of NTM, including *M. avium* complex, *M. intracellulare*, *M. abscessus*, *M. kansasii*, *M. malmoense*, *M. gordonae*, *M. szulgai*, *M. scrofulaceum*, *M. chelonae*, *M. neoaurum*, *M. asiaticum*, *M. phlei*, *M. triviale*, *M. gastri*, *M. fortuitum*, and *M. parafortuitum*. A sample was considered positive for NTM when the ROX channel concentration exceeded 2.5 copies/μL.

### Limit of detection of ddPCR assay

We tested the limit of detection (LOD), defined as the lowest concentration at which the target could be reliably detected, for the ddPCR assay in detecting both MTB and NTM. For MTB, the LOD was evaluated using inactivated BCG strains obtained from two sources [Fudan University (ATCC35734) and Hong Kong]. For NTM, the LOD was tested using *M. kansasii*, *M. scrofulaceum*, *M. szulgai*, *M. abscessus*, *M. neoaurum*, and *M. gordonae*. For each mycobacterial strain, six serial dilutions were prepared, and five replicates were tested at each concentration to establish the preliminary LOD. Subsequently, three concentrations near the preliminary LOD were selected, and 20 replicate tests were performed at each concentration to refine the estimate. The final LOD for each mycobacterium was determined through statistical analysis using SPSS.

### Statistical analysis

Categorical variables were reported as absolute frequencies and percentages. The concordance between Xpert and ddPCR was performed with Kappa test. *p* < 0.05 was considered as statistically significant. Data analyses were conducted using SPSS version 21.0 software (SPSS Inc., Chicago, IL, United States).

## Results

### Participant characteristics

**Figure 1:**
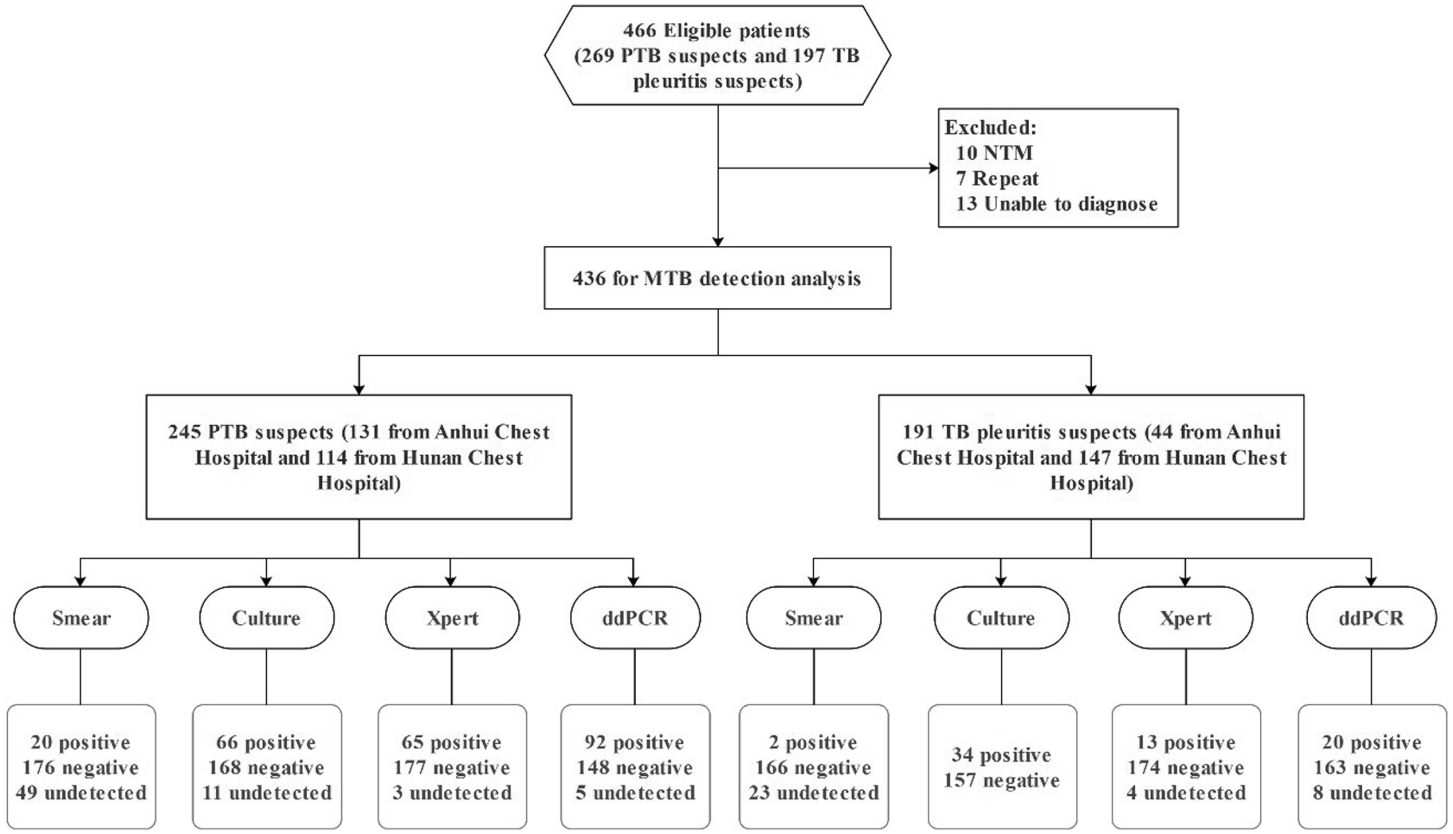
Patient inclusion and laboratory testing process.

A total of 466 patients with suspected PTB and TP were initially enrolled. After excluding 10 patients with NTM infection, 7 individuals with repeat results, and 13 patients with undetermined diagnosis, 436 patients were eligible for the final analysis, including 245 suspected PTB cases and 191 suspected TP cases (Figure 1). Of the patients, 308 (70.64%) were male and 123 (28.21%) were female, with the largest age group being 45–64 years (38.3%). Of the 245 suspected PTB patients, 170 (69.39%, 170/245) participants were confirmed as having PTB based on the final clinical diagnosis, among whom 66 (38.82%, 66/170) cases were culture positive. Among the 191 suspected TP patients, 142 (74.35%, 142/191) cases were identified as TP, with 34 (23.94%, 34/142) cases culture positive (Table 1 and Figure 1).

**Table 1:** General information of patients initially diagnosed with PTB and TP.

Baseline variable	Total (*N* = 436)	Number of PTB suspects (%)			Number of TP suspects (%)		
		Anhui Chest Hospital (*N* = 131)	Hunan Chest Hospital (*N* = 114)	All PTB suspects (*N* = 245)	Anhui Chest Hospital (*N* = 44)	Hunan Chest Hospital (*N* = 147)	All TP suspects (*N* = 191)
Sex							
Male	308 (70.64)	91 (69.47)	78 (68.42)	169 (68.98)	32 (72.73)	107 (72.79)	139 (72.77)
Female	123 (28.21)	40 (30.53)	32 (28.07)	72 (29.39)	12 (27.27)	39 (26.53)	51 (26.70)
Unknown	5 (1.15)	0	4 (3.51)	4 (1.63)	0	1 (0.68)	1 (0.52)
Age							
<25	45 (10.32)	13 (9.92)	12 (10.53)	15 (10.20)	5 (11.36)	15 (10.20)	20 (10.47)
25–44	73 (16.74)	24 (18.32)	19 (16.67)	43 (17.55)	10 (22.73)	20 (13.61)	30 (15.71)
45–64	167 (38.30)	51 (38.93)	48 (42.11)	99 (40.41)	13 (29.55)	55 (37.41)	68 (35.60)
≥65	147 (33.72)	43 (32.82)	32 (28.07)	75 (30.41)	16 (36.36)	56 (38.10)	72 (37.70)
Unknown	4 (0.92)	0 (0)	3 (2.63)	3 (1.22)	0	1 (0.68)	1 (0.52)
Sample type							
Bronchoalveolar lavage fluid	15 (3.44)	15 (11.45)	0	15 (6.12)	—	—	—
Sputum	230 (52.75)	116 (88.55)	114 (100)	230 (93.88)	—	—	—
Pleural effusion	191 (43.81)	—	—	—	44 (100)	147 (100)	191 (100)
Clinical diagnosis							
Positive	312 (71.56)	89 (67.94)	81 (71.05)	170 (69.39)	29 (65.91)	113 (76.87)	142 (74.35)
Negative	124 (28.44)	42 (32.06)	33 (28.95)	75 (30.61)	15 (34.09)	34 (23.13)	49 (25.65)

### Limit of detection

The limit of detection of ddPCR for BCG was 0.89 CFU/mL (Supplementary Figure S1). For NTM, the LOD varied substantially across different species. For example, the LOD for *M. kansasii* was 11.82 CFU/mL, whereas the LOD for *M. neoaurum* was 359.4 CFU/mL (Supplementary Figure S1). These findings indicate that while ddPCR demonstrates high sensitivity for detecting *Mycobacterium tuberculosis* complex, its sensitivity for NTM detection is comparatively lower and species-dependent.

### Performance of ddPCR assay against Xpert in PTB or TP based on final clinical diagnosis

**Figure 2:**
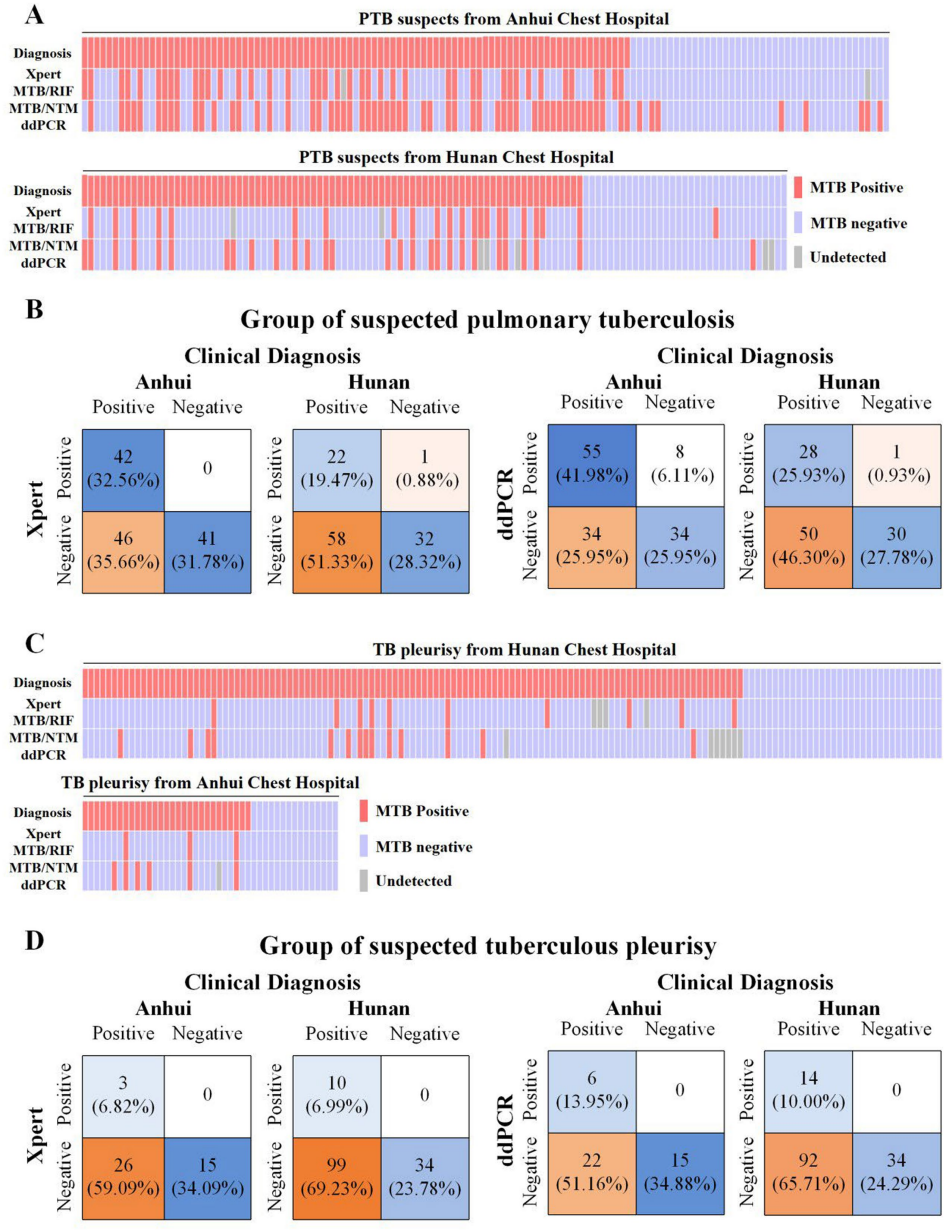
Performance of ddPCR and Xpert for TB detection compared with clinical diagnosis. **(A)** Individual patient-level results in the suspected PTB group. Each column represents one patient. Red: positive; blue: negative; gray: not tested. Results are shown for clinical diagnosis, Xpert, and ddPCR, stratified by enrollment site (Anhui Chest Hospital and Hunan Chest Hospital). **(B)** Comparison of Xpert and ddPCR against clinical diagnosis in suspected PTB cases. Numbers and percentages indicate true positives, false positives, true negatives, and false negatives for each assay at both sites. **(C)** Individual patient-level results in the suspected TP group, presented in the same format as **(A)**. **(D)** Comparison of Xpert and ddPCR against clinical diagnosis in suspected TP cases, presented in the same format as **(B)**.

To measure the sensitivity and specificity of ddPCR based assays against Xpert, we assessed their diagnostic performance with the final clinical diagnosis (Figure 2 and Supplementary Table S2). The sensitivity of ddPCR in suspected PTB was 49.70% (83/167; 95% CI: 42.12–57.31) [61.80% (55/89) in Anhui, 35.90% (28/78) in Hunan, respectively], which was significantly higher than that of Xpert 38.10% (64/168; 95% CI: 30.90–45.86) [47.73% (42/88) in Anhui, 27.50% (22/80) in Hunan, respectively] (*p* < 0.001). But the specificity of Xpert [98.65% (73/74; 95% CI: 92.78–99.80); 100% (41/41) in Anhui, 96.97% (32/33) in Hunan] in suspected PTB was higher than that of ddPCR [87.70% (64/73; 95% CI: 78.14–93.36); 80.95% (34/42) in Anhui, 96.77% (30/31) in Hunan] (*p* > 0.05).

For suspected TP group, the sensitivity of ddPCR was 17.54% (20/114; 95% CI: 11.62–25.64) [21.43% (6/28) in Anhui, 13.21% (14/106) in Hunan, respectively], which was significantly higher than that of Xpert 10.40% (13/125; 95% CI: 6.16–17.02) [10.34% (3/29) in Anhui, 9.17% (10/109) in Hunan, respectively] (*p* < 0.001). Both ddPCR and Xpert assay had a specificity of 100% in Anhui and Hunan. The agreement between Xpert and ddPCR was moderate among PTB patients (*κ* = 0.530) and TP patients (*κ* = 0.435) (Figure 2 and Supplementary Table S2).

### Performance of ddPCR assay against Xpert in PTB or TP based on culture results

Based on culture results as the reference standard, ddPCR demonstrated significantly higher sensitivity than Xpert in both suspected PTB and TP groups, whereas Xpert exhibited superior specificity. In the suspected PTB group, the sensitivity of ddPCR was 81.54% (53/65; 95% CI: 70.45–89.09), compared to 75.00% (48/64; 95% CI: 63.19–84.06) for Xpert (*p* < 0.001). Subgroup analysis by region revealed similar trends, with ddPCR sensitivity reaching 85.37% (35/41) in Anhui versus 77.50% (31/40) for Xpert, and 75.00% (18/24) in Hunan versus 70.83% (17/24) for Xpert. Conversely, the specificity of Xpert was significantly higher than that of ddPCR [91.52% (151/165) vs. 78.79% (130/165); *p* < 0.001]. This pattern persisted across both regions, with Xpert specificity of 87.64% (78/89) in Anhui and 96.05% (73/76) in Hunan, compared to 68.89% (62/90) and 90.67% (68/75) for ddPCR, respectively.

**Figure 3:**
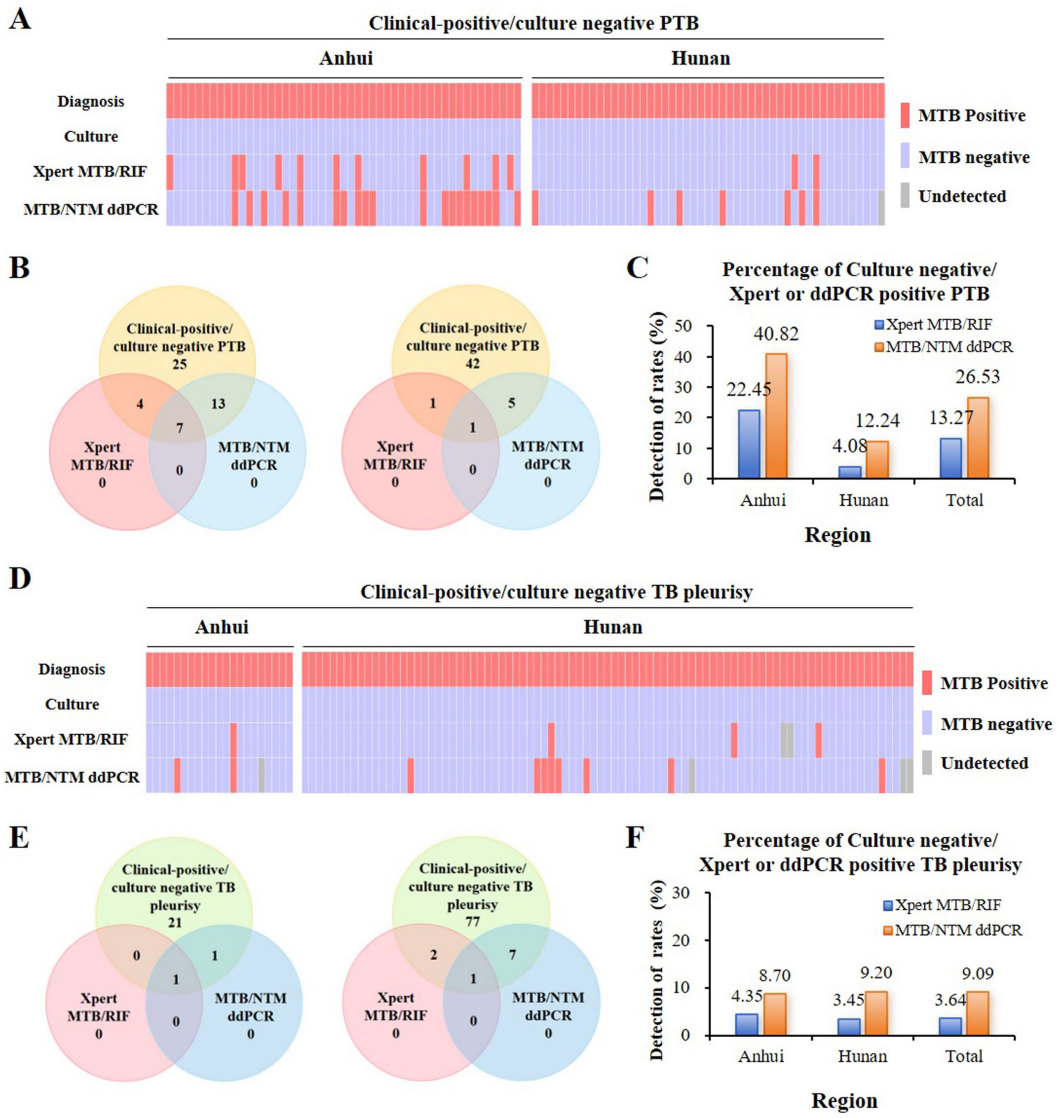
Performance of ddPCR and Xpert for TB detection compared with culture results. Individual patient-level results for culture-negative cases in the suspected PTB group **(A)** and suspected TP group **(D)**. Culture results (used as the reference standard) are shown alongside Xpert and ddPCR outcomes for each patient. Each column represents one patient. Red: positive; blue: negative; gray: not tested. Bar charts showing the absolute numbers of culture-negative but Xpert- or ddPCR-positive cases in the PTB group **(B)** and TP group **(E)**, with results displayed separately for the Anhui and Hunan sites. The stacked bars also indicate the number of cases positive by both molecular assays. Stacked bar charts presenting the proportion (%) of culture-negative cases that were detected by Xpert or ddPCR in the PTB group **(C)** and TP group **(F)**.

In the suspected TP group, ddPCR also outperformed Xpert in sensitivity, with values of 33.33% (10/30) versus 28.13% (9/32) (*p* = 0.006). Regional analysis showed ddPCR sensitivity of 50.00% (4/8) in Anhui and 27.30% (6/22) in Hunan, while Xpert demonstrated 25.00% (2/8) and 29.17% (7/24), respectively. However, Xpert maintained significantly higher specificity than ddPCR [97.42% (151/155; 95% CI: 93.57–99.03) vs. 93.46% (143/153; 95% CI: 88.32–96.48); *p* < 0.001]. Regional specificity for Xpert was 97.22% (35/36) in Anhui and 97.48% (116/119) in Hunan, compared to 94.29% (33/35) and 93.22% (110/118) for ddPCR. Agreement between the two assays was moderate among culture-positive PTB patients (*κ* = 0.596), but only fair among culture-positive TP patients (*κ* = 0.279) (Figure 3 and Supplementary Table S3).

### Efficacy in identifying NTM infection for ddPCR

**Figure 4:**
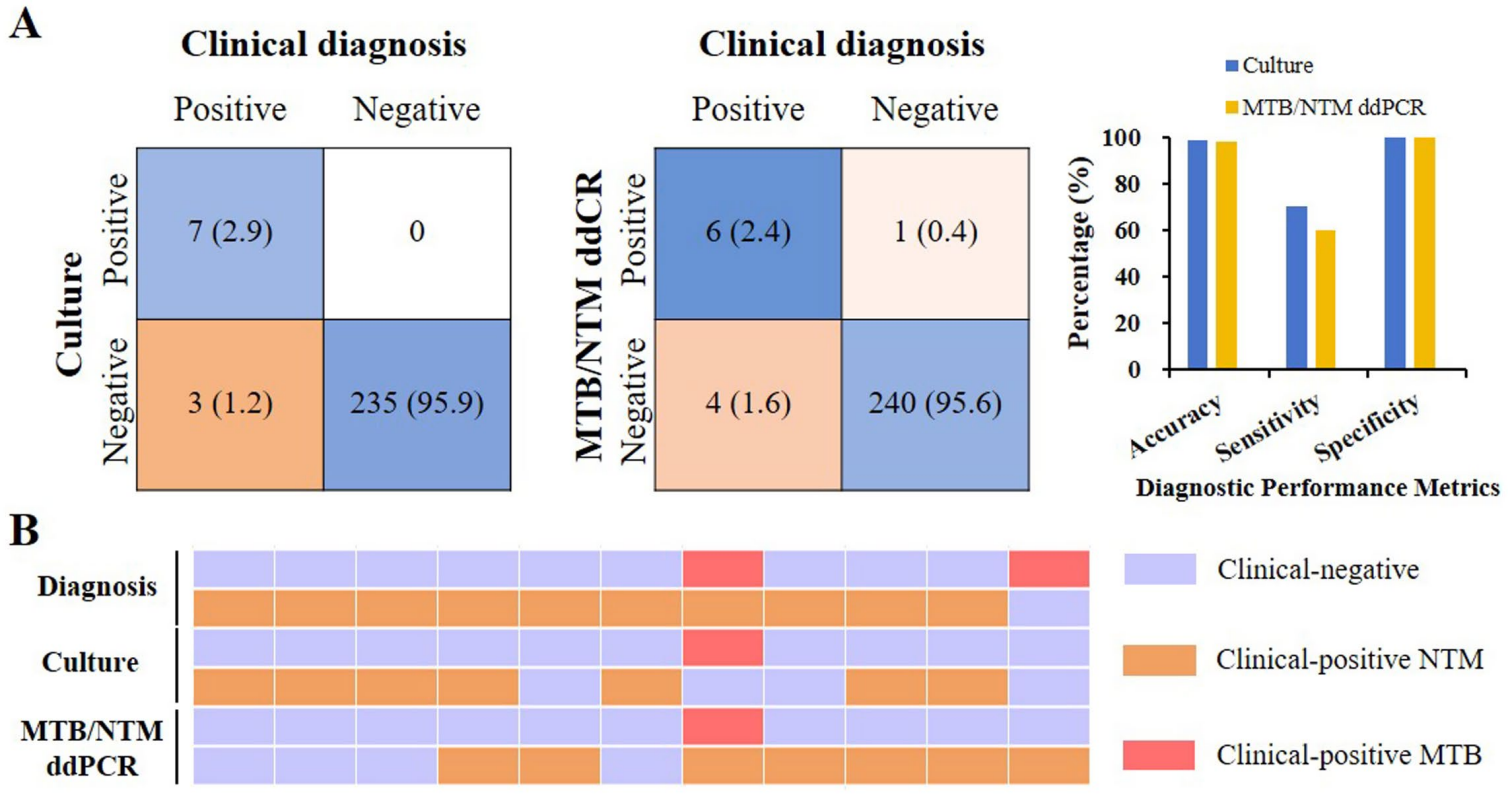
The ddPCR’s detection efficacy in identifying NTM/MTB. **(A)** Sensitivity, specificity, and accuracy statistics of culture and ddPCR detection in detecting NTM. **(B)** In culture-negative or tuberculosis-positive patients, ddPCR can help to detect the NTM infection.

Of the 10 individuals with NTM infection, 7 (70%) cases were culture positive, 6 (60%) cases were ddPCR positive. The specificity in identifying NTM infection was 100% (235/235) for culture, and 99.58% (236/237) for ddPCR, respectively. And the accuracy was 98.78% (242/245) and 97.98% (242/247) for culture and ddPCR, respectively. In addition, all three cases of culture-negative were confirmed as NTM infection by ddPCR (Figure 4).

## Discussion

Conventional diagnostic methods are limited by suboptimal sensitivity and prolonged turnaround times (16), highlighting the urgent need for novel high-sensitivity approaches such as ddPCR. This technique has proven particularly valuable for detecting tuberculosis in paucibacillary samples with low bacterial abundance (17). However, no study has reported to confirm the diagnostic value of ddPCR for PTB and TP, particularly for paucibacillary culture-negative PTB and TP in a large number of clinical samples.

*M. tuberculosis*-specific targets are crucial for ddPCR analysis. The *IS6110*, a specific and conserved sequence in MTB, was commonly used as a target for detecting the presence of MTB (13, 18, 19). Moreover, *23S rRNA* also provided a target for the rapid detection and identification of mycobacteria both at the genus and species levels (20, 21). Due to a single copy or the lack of *IS6110* sequence in a few MTB, *23S rRNA* is used as a supplementary target for the detection of MTB (22–24). Therefore, we selected *IS6110* and *23S rRNA* as the detection targets in this study to reduce false-negative results and improve the sensitivity of detection.

It has been reported that the sensitivity of *IS6110* ddPCR, targeting two different locations in the *IS6110* sequence, outperformed the Xpert assay (61.60% vs. 38.06%) in confirmed PTB patients (25). Though there were differences in results between the two hospitals for ddPCR, which may be attributed to variations in sample size, the influence of patients’ immune status and comorbidities, and the disease state of the patients (e.g., early/mild PTB or cavitary PTB), we found that ddPCR showed a higher sensitivity than Xpert in suspected PTB based on final diagnosis or culture results.

However, the specificity of ddPCR was consistently lower than that of Xpert across both PTB and TP groups, a finding that warrants careful consideration. Mechanistically, this can be attributed to several factors. First, ddPCR amplifies nucleic acids regardless of bacterial viability, making it prone to detecting residual DNA from previously treated or inactive infections, leading to false-positive results in patients without active disease. Second, the ultra-high sensitivity of ddPCR increases the risk of detecting trace environmental or laboratory contamination. Given the ubiquitous presence of mycobacterial DNA in laboratory settings and the potential for cross-contamination during sample processing, even minimal contamination can be amplified above the positivity threshold. Third, suboptimal sample preparation, variable DNA extraction efficiency, the presence of PCR inhibitors, and uneven distribution of *M. tuberculosis* in clinical specimens may also contribute to false-positive outcomes. Beyond mechanistic considerations, technical limitations inherent to ddPCR warrant discussion. Although ddPCR offers absolute quantification without standard curves, the precise assignment of positivity thresholds remains challenging. The cutoff values used in this study (0.46 copies/μL for MTB and 2.5 copies/μL for NTM) were derived from preliminary experiments and may not be universally applicable across different populations or specimen types. Moreover, the performance of ddPCR is influenced by sample quality, as specimens with low cellularity or high levels of PCR inhibitors can yield unreliable results.

The superior sensitivity of ddPCR makes it particularly valuable as a confirmatory test in patients with a high clinical suspicion of tuberculosis, especially those with smear-negative or culture-negative disease, where conventional methods often fail to establish a diagnosis. In such cases, a positive ddPCR result can prompt timely initiation of anti-tuberculosis treatment. Conversely, the lower specificity necessitates caution in interpreting positive results, particularly in low-prevalence settings or in patients with a history of prior tuberculosis, where residual DNA from previous infection may persist. Given the potential for false positives, a positive ddPCR result should ideally be confirmed by an alternative method, such as culture or a second molecular assay, especially when clinical suspicion is low.

Moreover, previous report showed that ddPCR, targeting *IS6110* and *IS1081*, had a higher sensitivity than Xpert (57.6% versus 23.0%) in patients with TP (26). And the ddPCR assay developed in this study confirmed that it is more sensitive than Xpert in suspected TP based on final diagnosis or culture results, demonstrating that ddPCR method has the potential to accurately quantify MTB pathogen with a low abundance. However, despite its improved performance, the sensitivity of ddPCR in tuberculous pleurisy remains relatively limited. This is primarily attributable to the paucibacillary nature of pleural TB, where the bacterial burden in pleural fluid is extremely low, often falling below the detection threshold even for highly sensitive molecular assays. The pathogenic mechanism of pleural TB is largely driven by a delayed hypersensitivity reaction to MTB antigens, rather than direct bacterial invasion of the pleural space, which further explains the low yield of pathogen-based detection methods. Given these limitations, alternative diagnostic modalities should be considered to complement ddPCR in clinical practice. Adenosine deaminase (ADA) testing remains a well-established, cost-effective biomarker with high sensitivity for pleural TB. While pleural biopsy, either via closed needle or thoracoscopic approaches, provides histopathological confirmation and culture material, representing the diagnostic reference standard in many settings. Integrating ddPCR with these complementary methods may enhance overall diagnostic yield in suspected tuberculous pleurisy.

Although pulmonary disease caused by NTM shares many clinical features with tuberculosis, the therapeutic strategies differ substantially (27, 28). Therefore, accurate differentiation between MTB and NTM infection is of critical importance. However, conventional culture-based methods are time-consuming and require species-specific culture conditions. Moreover, culture typically detects a single organism; in the context of MTB infection, concurrent NTM infection may be missed. In contrast, the MTB/NTM ddPCR assay described herein is capable of simultaneously detecting MTB and more than 10 clinically relevant NTM species, including *M. intracellulare*, *M. kansasii*, and *M. abscessus*. This dual-target design confers a clear advantage over culture techniques in terms of throughput and turnaround time, although culture remains indispensable for obtaining isolates for drug susceptibility testing. Notably, while the sensitivity and accuracy of MTB/NTM ddPCR were slightly lower than those of culture, its ability to detect co-infections represents a distinct clinical benefit. In one instance, the assay correctly identified a patient with concurrent MTB and NTM infection, whereas culture detected only MTB. Furthermore, all three culture-negative but clinically diagnosed cases were confirmed as NTM infection by ddPCR. Among the four culture-positive but ddPCR-negative NTM cases, the isolates were identified as *M. kansasii*, *M. avium*, and other rare NTM species. These discrepancies may be attributable to mixed infections or contamination, underscoring the need for comprehensive interpretation integrating clinical, microbiological, and radiological findings. In summary, MTB/NTM ddPCR serves as a valuable adjunct to culture-based methods for the identification of mycobacterial species, offering enhanced detection of co-infections and culture-negative cases.

This study has several limitations that should be considered when interpreting the findings. First, this study was conducted at only two hospitals in China, which may limit the generalizability of the findings. The geographic restriction means that the results may not be representative of broader populations across China or other regions with different tuberculosis epidemiology, healthcare infrastructures, or patient demographics. Future multicenter studies incorporating diverse geographic settings are needed to validate the applicability of our findings. Second, the number of patients with NTM infection was small, which limits the robustness of conclusions regarding ddPCR performance for NTM detection. With such a limited sample, we were unable to perform stratified analyses by NTM species. Further large-scale studies are required to characterize the specificity of ddPCR across the full spectrum of clinically relevant NTM species. Third, important clinical variables, including HIV status, immunosuppressive therapy, and other comorbidities, were not systematically collected. These factors are known to influence both the likelihood of tuberculosis infection and the performance of molecular diagnostic assays. The absence of this information precludes subgroup analyses that could have elucidated whether ddPCR performance varies according to immune status, which is particularly relevant given that immunocompromised patients often present with paucibacillary disease. Future studies should incorporate comprehensive clinical characterization to better define the utility of ddPCR in specific patient subgroups. Fourth, the ddPCR assay lacked external validation in an independent cohort. While our results demonstrate promising diagnostic performance, the absence of validation in a separate population raises the possibility of overfitting to the current dataset. The cutoff values used in this study were derived from preliminary experiments and may require adjustment when applied to different populations or specimen types. Independent validation studies are essential to confirm the reproducibility and generalizability of our findings. Fifth, the study did not systematically evaluate the impact of pre-analytical variables, such as specimen storage conditions, transport time, and DNA extraction methods, on ddPCR performance. Variations in these factors could contribute to the observed variability in results and may affect the reproducibility of the assay across different laboratory settings. Despite these limitations, our study provides valuable insights into the diagnostic performance of ddPCR for PTB and TP, particularly in paucibacillary and culture-negative cases. However, the findings should be considered preliminary, and larger, more comprehensive studies are warranted to confirm and extend these observations.

## Conclusion

This study demonstrates that ddPCR is a highly sensitive and accurate molecular assay for the diagnosis of PTB and TP, particularly in paucibacillary and culture-negative cases. From a clinical perspective, ddPCR offers a valuable adjunct to conventional microbiological tests. Its high sensitivity makes it particularly useful for patients with suspected PTB or TP who are smear or culture-negative, enabling earlier diagnosis and timely initiation of anti-tuberculosis treatment. In addition, the ability to simultaneously detect MTB and NTM provides important guidance for appropriate antimicrobial therapy, avoiding unnecessary anti-TB treatment in NTM patients.

Future research should focus on validating these findings in larger, multicenter cohorts, particularly in pediatric populations and immunocompromised patients, where bacterial loads are typically low. Furthermore, standardization of ddPCR protocols and interpretation criteria will be essential to facilitate its broader clinical application.

## Data Availability

The original contributions presented in the study are included in the article/Supplementary material, further inquiries can be directed to the corresponding authors.
