# Supplementary material for: Evaluation of a new droplet digital PCR for diagnosis of pulmonary tuberculosis and tuberculous pleurisy
Source: Front Med (Lausanne). 2026 Apr 15;13:1819767. doi: 10.3389/fmed.2026.1819767 (PMC13124612; doi:10.3389/fmed.2026.1819767)
Supplement: Supplementary file 1 [file Table_1.DOCX]

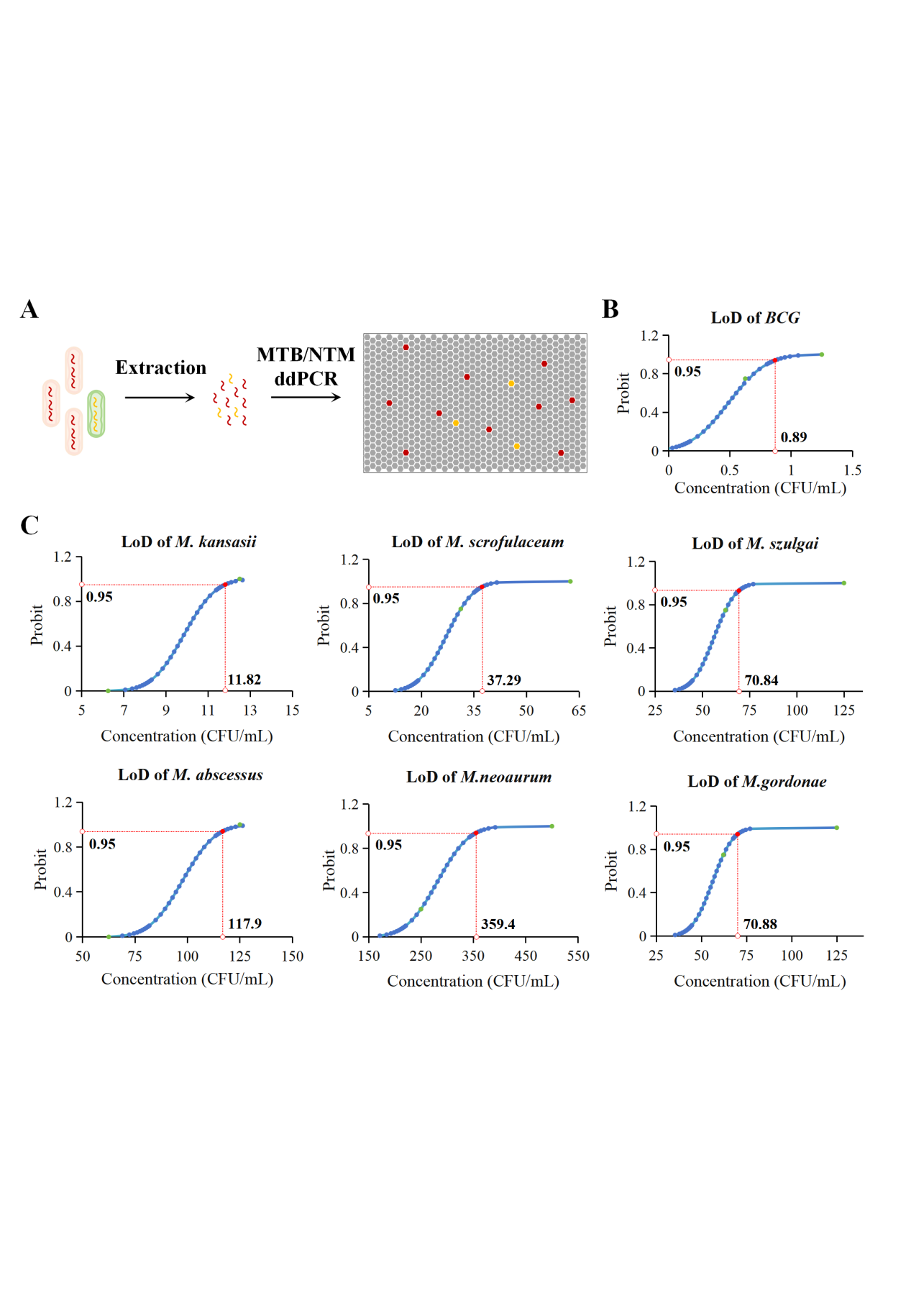


Figure S1. ddPCR in detecting NTM/MTB. A. The detection principle of ddPCR; B. The LOD of

ddPCR for detecting BCG; C. The LOD of ddPCR for detecting NTM varies in different NTM

strains.

Table S1. Composite reference standard for classification of tuberculosis status

| Category | Microbiological criteria | Radiological criteria | Clinical criteria | Treatment criteria | Final diagnosis |
| --- | --- | --- | --- | --- | --- |
| Bacteriologically confirmed TB | Any positive: culture/Xpert/smear | Any (may be present or absent) | Any | Any | Confirmed TB |
| Clinically diagnosed TB | All negative: culture/Xpert/smear | Consistent with active TB | Compatible with TB | Full course initiated | Clinical TB |
| Non-TB | All negative  (if performed) | Not consistent with TB, or alternative diagnosis | Not compatible with TB, or alternative diagnosis established | No anti-TB treatment, or alternative treatment initiated | Non-TB |

Table S2. The accuracy of Xpert and ddPCR for the diagnosis of PTB and TP based on final clinical diagnosis

| Group | Method | Sensitivity, % (n/N) | 95% CI | *p* | Specificity, % (n/N) | 95% CI | *p* | Xpert/ddPCR Kappa |
| --- | --- | --- | --- | --- | --- | --- | --- | --- |
| PTB | Xpert | 38.10% (64/168) | 30.90–45.86 | ＜0.001 | 98.65% (73/74) | 92.78–99.80 | 0.718 | 0.530 |
|  | ddPCR | 49.70% (83/167) | 42.12–57.31 |  | 87.70% (64/73) | 78.14–93.36 |  |  |
| TP | Xpert | 10.40% (13/125) | 6.16–17.02 | ＜0.001 | 100%(49/49) | 92.75–100 | ＞0.05 | 0.435 |
|  | ddPCR | 17.54% (20/114) | 11.62–25.64 |  | 100%(49/49) | 92.75–100 |  |  |

PTB, pulmonary tuberculosis; TP, tuberculous pleurisy; CI, confidence interval; ddPCR, digital polymerase chain reaction

Table S3. The accuracy of Xpert and ddPCR for the diagnosis of PTB and TP based on culture results

| Group | Method | Sensitivity, % (n/N) | 95% CI | *p* | Specificity, % (n/N) | 95% CI | *p* | Xpert/ddPCR Kappa |
| --- | --- | --- | --- | --- | --- | --- | --- | --- |
| PTB | Xpert | 75.00% (48/64) | 63.19–84.06 | ＜0.001 | 91.52% (151/165) | 86.27–94.91 | ＜0.001 | 0.596 |
|  | ddPCR | 81.54% (53/65) | 70.45–89.09 |  | 78.79% (130/165) | 71.94–84.35 |  |  |
| TP | Xpert | 28.13% (9/32) | 15.53–45.51 | 0.006 | 97.42% (151/155) | 93.57–99.03 | ＜0.001 | 0.279 |
|  | ddPCR | 66.67% (20/30) | 48.78–80.86 |  | 93.46% (143/153) | 88.32–96.48 |  |  |

PTB, pulmonary tuberculosis; TP, tuberculous pleurisy; CI, confidence interval; ddPCR, digital polymerase chain reaction
